# Tacrolimus Reverses UVB Irradiation-Induced Epidermal Langerhans Cell Reduction by Inhibiting TNF-α Secretion in Keratinocytes via Regulation of NF-κB/p65

**DOI:** 10.3389/fphar.2018.00067

**Published:** 2018-02-22

**Authors:** JiaLi Xu, YaDong Feng, GuoXin Song, QiXing Gong, Li Yin, YingYing Hu, Dan Luo, ZhiQiang Yin

**Affiliations:** ^1^Department of Oncology, First Affiliated Hospital of Nanjing Medical University, Nanjing, China; ^2^Department of Gastroenterology, First Affiliated Hospital of Nanjing Medical University, Nanjing, China; ^3^Department of Gastroenterology, Zhongda Hospital, School of Medicine, Southeast University, Nanjing, China; ^4^Department of Pathology, First Affiliated Hospital of Nanjing Medical University, Nanjing, China; ^5^Department of Dermatology, First Affiliated Hospital of Nanjing Medical University, Nanjing, China; ^6^Department of Dermatology, Affiliated Wuxi People’s Hospital, Nanjing Medical University, Wuxi, China

**Keywords:** tacrolimus, UVB, Langerhans cells, TNF-α, NF-κB

## Abstract

**Background:** Topical calcineurin inhibitors including tacrolimus and pimecrolimus are used in the treatment of many inflammatory skin diseases mainly via blocking T-cell proliferation. Our previous studies found that pimecrolimus 1% cream could reverse high-dose ultraviolet B (UVB) irradiation-induced epidermal Langerhans cell (LC) reduction via inhibition of LC migration. We conducted this study to investigate the effects of topical tacrolimus 0.03% ointment on high-dose UVB-irradiated human epidermal LCs.

**Methods:** Twenty fresh human foreskin tissues were randomly divided into four groups as follows: *Control*, *Tacrolimus* (0.03%), *UVB* (180 mJ/cm^2^), *and UVB* (180 mJ/cm^2^) + *Tacrolimus* (0.03%). Four time points were set as follows: 0, 18, 24, and 48 h. We collected culture medium and tissues at each time point. The percentage of CD1a+ cells in the medium was detected by means of flow cytometry. Each tissue was prepared for immunohistochemistry, real-time quantitative PCR, and western blot. HaCaT cells were cultured and divided into four groups: *Control*, *Tacrolimus* (1 μg/ml), *UVB* (30 mJ/cm^2^), *and UVB* (30 mJ/cm^2^) + *Tacrolimus* (1 μg/ml). The cells were incubated for 24 h and prepared for real-time quantitative PCR and western blot.

**Results:** Topical tacrolimus significantly reversed high-dose UVB irradiation-induced epidermal LC reduction and CD1a+ cell increment in culture medium. Tacrolimus significantly inhibited UVB irradiation-induced tumor necrosis factor-α (TNF-α) and nuclear factor kappa B (NF-κB)/p65 mRNA and protein expression in HaCaT cells. Tacrolimus also significantly inhibited high-dose UVB irradiation-induced TNF-α expression in cultured tissues. Finally, TNF-α antagonist (recombinant human TNF-α receptor II: IgG Fc fusion protein) could significantly reverse UVB irradiation-induced epidermal LC reduction.

**Conclusion:** Topical tacrolimus 0.03% could reverse UVB irradiation-induced epidermal LC reduction by inhibiting TNF-α secretion in keratinocytes via regulation of NF-κB/p65.

## Introduction

Topical calcineurin inhibitors (TCIs), including tacrolimus ointment and pimecrolimus cream, are widely used in the treatment of atopic dermatitis and many other inflammatory skin diseases, where the central therapeutic mechanism is to block T-cell proliferation and inhibit the activation of T-cells and thereby diminish inflammation (Yin et al., 2014; Nygaard et al., 2017). In contrast with topical corticosteroids, TCIs are not related to atrophy or increased percutaneous absorption after long-term use and have much lower possibility for systemic effects (Siegfried et al., 2016).

There is no evidence that TCIs have direct action on epidermal Langerhans cells (LCs). Epidermal LCs have the action of immunosurveillance and process antigen and migrate to local draining lymphnodes from epidermis, expressing CD1a (Rowden, 1980). Single high-dose ultraviolet B (UVB) irradiation can induce significant epidermal LC depletion in human skin, and the increased migration of LCs might be the main mechanism (Kölgen et al., 2002). UVB-induced apoptotic cells are phagocytosed by LCs *ex vivo*, which has an important anti-inflammatory effect in the resolution of UVB-induced cutaneous inflammation (Hatakeyama et al., 2017).

Our previous studies found that high-dose UVB irradiation significantly decreased the number of epidermal LCs, and pimecrolimus 1% cream could reverse these changes via inhibition of LCs migration by regulation of tumor necrosis factor-α (TNF-α) and E-cadherin (Yin et al., 2012b, 2014). Whether topical tacrolimus (another kind of widely used TCI) can also inhibit LC migration in UVB-irradiated skin is still unclear.

Lan et al. (2005) reported UVB irradiation-induced secretion of TNF-α from keratinocytes, which could be inhibited by tacrolimus by downregulation of nuclear factor kappa B (NF-κB) expression. Wu et al. (2012) found that tacrolimus didn’t affect the nuclear activation and translocation of NF-κB/p50; however, UVB irradiation-induced NF-κB/p65 nuclear expression was suppressed by tacrolimus. This study aimed to investigate the effect of topical tacrolimus 0.03% ointment on high-dose UVB-irradiated human epidermal LCs and the possible relation with TNF-α secretion and NF-kB/p65 regulation, which would contribute to further understanding of the mechanism of TCI action and treatment.

## Materials and Methods

### Ethics Statement

This study was carried out in accordance with the recommendations of institutional guidelines and Local Ethics Committee of the First Affiliated Hospital of Nanjing Medical University (approval number 2013-SRFA-074). Written informed consent was obtained from all subjects.

### Study Design

Twenty fresh human foreskin tissues were obtained from Department of Urology by circumcision, consented by the patients (age range 18–30 years). All subjects gave written informed consent in accordance with the Declaration of Helsinki. The tissues were randomly divided into four groups of five each, as follows: *Control*, *Tacrolimus* (tissues were applied once with topical tacrolimus 0.03% on the epidermis), *UVB* (tissues were irradiated once with 180 mJ/cm^2^ UVB on the epidermis), and *UVB + Tacrolimus* (tissues were applied on the epidermis with topical tacrolimus 0.03% after 180 mJ/cm^2^ UVB irradiation). The tissues were processed and cultured as previously described (Yin et al., 2012b). Recombinant human TNF-α receptor II: IgG Fc fusion protein (Yisaipu; CP Guojian Pharmaceutical Co., Ltd., Shanghai, China; 50 μg/ml) was added into culture medium to block the effect of TNF-α.

The UVB source was a BLE-1T158 UV lamp (Spectronics Corp., Westbury, NY, United States) by which 180 mJ/cm^2^ UVB was delivered once to the epidermis. After UVB irradiation or not, tacrolimus 0.03% ointment (Protopic; Astellas Toyama Co., Toyama, Japan) was applied on the epidermis. Ten minutes after application or irradiation, 1 ml culture medium was added to each well to immerse the whole tissue. All tissues were cultured at 37°C.

Four time points were set as follows: 0, 18, 24, and 48 h. For each group, each tissue was cut into four pieces corresponding to four time points. We collected culture medium and tissues at each time point, following which each tissue was cut into three parts. The percentage of CD1a+ cells in the medium was detected by means of flow cytometry. Each tissue was prepared for immunohistochemistry, real-time quantitative PCR, and western blot.

Keratinocyte line HaCaT cells were cultured as previously described (Zhou et al., 2013), and seeded in 12-well culture plates and divided into four groups, as follows: *Control*, *Tacrolimus* (Prograf; Astellas Ireland Co., Ltd., Killorglin, Co. Kerry, Ireland; 1 μg/ml), *UVB* (30 mJ/cm^2^), and *UVB* (30 mJ/cm^2^) + *Tacrolimus* (1 μg/ml). The cells were incubated for 24 h, and then prepared for real-time quantitative PCR and western blot. Experiments were repeated independently at least three times.

### Flow Cytometry

Detection of CD1a expression on cells in the culture medium was performed using anti-human CD1a-PE antibody (BioLegend, Inc., San Diego, CA, United States) (Yin et al., 2014). A FACS Calibur^TM^ Flow Cytometer (BD Biosciences, Franklin Lakes, NJ, United States) was used to gather data and images.

### Immunohistochemistry

Slides were prepared using a Ventana autoimmunostainer (Loche, United States) and available CD1a monoclonal antibody (Maixin-Bio, Fuzhou, Fujian, China) and active caspase-3 polyclonal antibody (Abcam, New Territories, Hong Kong, China). Detection utilized Polymer-HRP, with 3,3′-diaminobenzidine chromogen, and slides were visualized at 40× with a Nikon Eclipse microscope (Yin et al., 2012b). The number of typical CD1a positive epidermal LCs was counted for five successive fields in high magnification (HM, 400×). The number of LCs was calculated and expressed as CD1a+ LC/HM.

### Real-Time Quantitative PCR

Total mRNA was extracted from part (50 mg) of the aforementioned collected tissue, using TRIzol^®^ Reagent (Invitrogen; Life Technologies Corp., Carlsbad, CA, United States). The HaCaT cells attached to the culture plates were washed three times using PBS and then dissolved in TRIzol^®^ Reagent. First-strand cDNA was synthesized from 2 μg of total RNA. PCR amplification was performed in a total volume of 20 μl containing 1 μl template cDNA and 10 μl Real-Time PCR Master Mix (SYBR Green) (TOYOBO, Japan), and transcripts quantified using StepOnePlus^TM^ Real-Time PCR System (Applied Biosystems, United States) (Yin et al., 2017). All values were normalized to the expression of glyceraldehyde phosphate dehydrogenase (GAPDH). Primer sequences are shown in **Table 1**.

**Table 1:** Primers for the target genes in real-time quantitative PCR.

Target genes	Primers
GAPDH (115 bp)	Sense: 5′-CATCTTCTTTTGCGTCGCCA-3′
	Antisense: 5′-TTAAAAGCAGCCCTGGTGACC-3′
IL-18 (77 bp)	Sense: 5′-GGGAAGAGGAAAGGAACCTC-3′
	Antisense: 5′-CCATCTTTATTCCTGCGACA-3′
TNF-α (66 bp)	Sense: 5′-CCTCTTCTCCTTCCTGATCG-3′
	Antisense: 5′-ATCACTCCAAAGTGCAGCAG-3′
E-cadherin (142 bp)	Sense: 5′-CAGCGTGTGTGACTGTGAAG-3′
	Antisense: 5′-AAACAGCAAGAGCAGCAGAA-3′
NF-κB/p65 (142 bp)	Sense: 5′-GCATCCAGACCAACAACAAC-3′
	Antisense: 5′-ATGGGATGAGAAAGGACAGG-3′
IL-1β (115 bp)	Sense: 5′-AAGCTGAGGAAGATGCTGGT-3′
	Antisense: 5′-CGTTATCCCATGTGTCGAAG-3′
CCR7 (141 bp)	Sense: 5′-GGGAGAGTGTGGTGTTTCCT-3′
	Antisense: 5′-CCTGACATTTCCCTTGTCCT-3′
CCL19 (83 bp)	Sense: 5′-AAGACTGCTGCCTGTCTGTG-3′
	Antisense: 5′-GCCATCCTTGATGAGAAGGT-3′
MMP-9(81 bp)	Sense: 5′-CCGGACCAAGGATACAGTTT-3′
	Antisense: 5′-CGGCACTGAGGAATGATCTA-3′
ITGA6 (101 bp)	Sense: 5′-AACTGGAAAGGGATTGTTCG-3′
	Antisense: 5′-TGCTCAGTCTCTCCACCAAC-3′

*Abbreviation: GAPDH, glyceraldehyde phosphate dehydrogenase; IL-18, interleukin-18; TNF-α, tumor necrosis factor-α; NF-κB, nuclear factor kappa B; IL-1β, Interleukin-1β; CCR7, C–C chemokine receptor type 7; CCL19, C–C motif ligand 19; MMP-9, matrix metallopeptidase 9; ITGA6, integrin alpha-6*.

### Western Blot

The collected tissues and HaCaT cells were homogenized in cold lysis buffer containing protease inhibitor. Centrifugal separation was conducted at 4°C, at 14,000 rpm for 15 min. The upper layer of the solution was tested for protein using the Bradford method. SDS–PAGE was performed (Yin et al., 2017). The primary antibody was added as below: TNF-α, β-actin (Biosynthesis Bio, Beijing, China), and NF-κB/p65 (KeyGEN Biotech, Nanjing, Jiangsu, China), following the manufacturer’s instructions. Differences in protein expression were examined using Gel-Pro 32 (Media Cybernetics, Rockville, MD, United States).

### Statistical Analysis

Data analysis was conducted by using GraphPad Prism for Windows (GraphPad Software, San Diego, CA, United States). All data were presented as mean ±*SD*. Data were tested for normality and statistical significance calculated using a Student’s *t*-test, Mann–Whitney *U*-test, or Friedman’s test, as appropriate (Yin et al., 2017). *P* < 0.05 was considered statistically significant.

## Results

### Topical Tacrolimus Reverses High-Dose UVB Irradiation-Induced Epidermal Langerhans Cell Reduction

**FIGURE 1:**
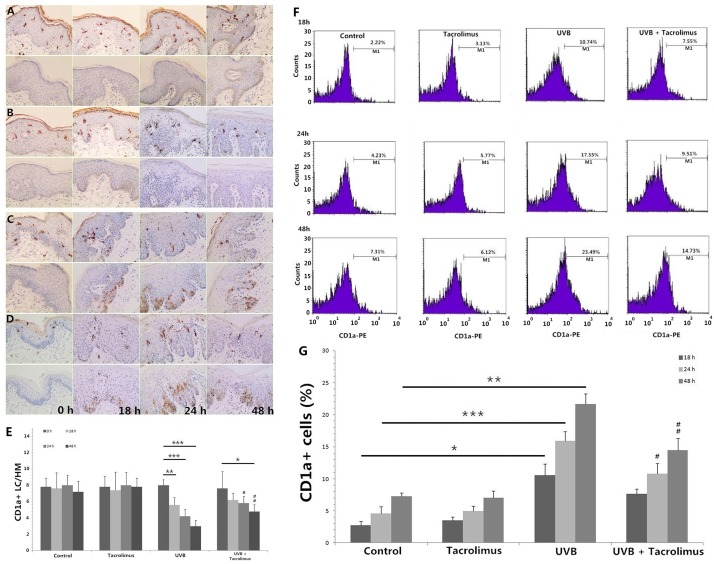
Immunohistochemical staining of CD1a and active caspase 3 in foreskin tissues (**A–E**, HM × 400) and flow cytometry of CD1a expression on cells in the culture medium **(F,G)** at different time points. **(A)** *Control* group, **(B)** *Tacrolimus* group, **(C)** *UVB* group, **(D)** *UVB + Tacrolimus* group, **(E)** Topical tacrolimus significantly reversed 180 mJ/cm^2^ UVB irradiation-induced marked epidermal CD1a+ LC reduction in tissues at 24 and 48 h, **(F)** Flow cytometry of CD1a, **(G)** Topical tacrolimus significantly inhibited UVB irradiation-induced marked CD1a+ cell increment in the culture medium at 24 and 48 h. Statistical significance indicated: ^∗^*P* < 0.05, ^∗∗^*P* < 0.01, ^∗∗∗^*P* < 0.001, ^#^*P* < 0.05, ^##^*P* < 0.01, ^###^*P* < 0.001. Abbreviation: UVB, ultraviolet B; LCs, Langerhans cells; HM, high magnification.

Immunohistochemistry showed that the number of epidermal CD1a+ LCs had no significant differences at different time points, for *Control* and *Tacrolimus* group. One hundred and eighty millijoules per centimeter squared UVB irradiation induced significant CD1a+ LC reduction at 18, 24, and 48 h (*P* < 0.01, respectively), which could be significantly reversed by topical tacrolimus 0.03% at 24 and 48 h (*P* < 0.05 and *P* < 0.01) (**Figures 1A–D**, upper panel, **E**).

The lower panel of **Figures 1A,B** show there were no obvious active caspase 3-positive cells in the epidermis for *Control* and *Tacrolimus* group. High-dose UVB irradiation induced obvious cell apoptosis in stratum spinosum and basal layer of epidermis, and *UVB + Tacrolimus* group seemed to have no visible difference in comparison with *UVB* group (**Figures 1C,D**, lower panel).

### Topical Tacrolimus Reverses High-Dose UVB Irradiation-Induced CD1a+ Cell Increment in Culture Medium

Flow cytometry (**Figures 1F,G**) showed 180 mJ/cm^2^ UVB irradiation induced significant increment of the percentage of CD1a+ cells in culture medium at 18, 24, and 48 h (18 h, *P* < 0.05; 24 h, *P* < 0.001; 48 h, *P* < 0.01), compared with *Control* group. Topical tacrolimus had no direct effect on the percentage of CD1a+ cells in culture medium; however, it could reverse UVB irradiation-induced CD1a+ cell increment (24 h, *P* < 0.05; 48 h, *P* < 0.01).

### Tacrolimus Inhibits UVB Irradiation-Induced TNF-α and NF-κB/p65 Expression in HaCaT Cells

**FIGURE 2:**
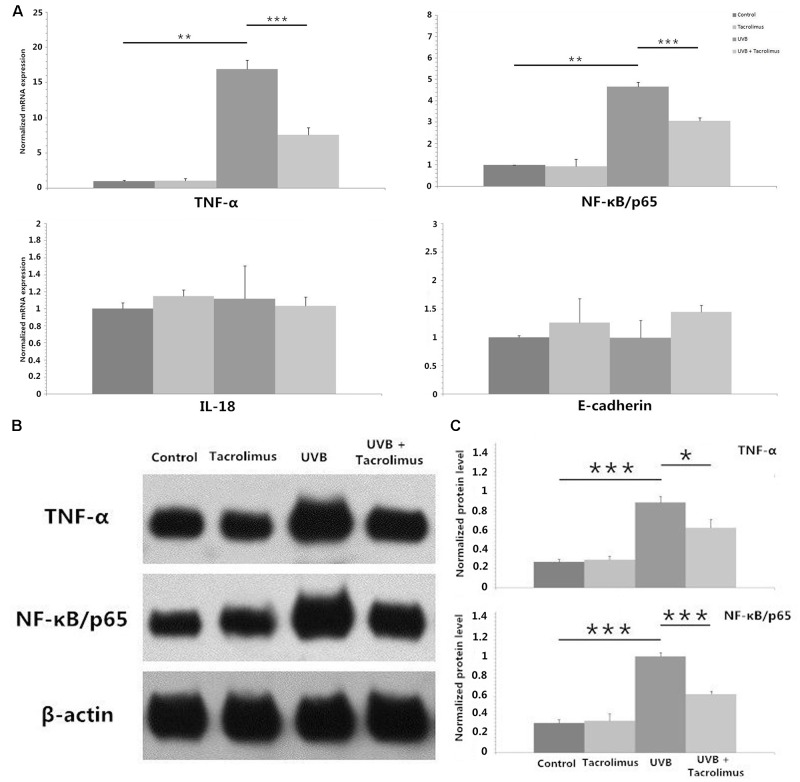
Real-time quantitative PCR analyses of TNF-α, NF-κB/p65, IL-18, and E-cadherin mRNA expression in HaCaT cells **(A)** and western blot of TNF-α and NF-κB/p65 protein expression in HaCaT cells **(B,C)** after 24 h of incubation. **(A)** One microgram per milliliter tacrolimus significantly inhibited 30 mJ/cm^2^ UVB irradiation-induced marked TNF-α and NF-κB/p65 mRNA expression. **(B)** Western blot of TNF-α and NF-κB/p65. **(C)** The inhibition of tacrolimus on UVB irradiation-induced TNF-α and NF-κB/p65 mRNA expression was confirmed at the protein level. Statistical significance indicated: ^∗^*P* < 0.05, ^∗∗^*P* < 0.01, ^∗∗∗^*P* < 0.001. Abbreviation: UVB, ultraviolet B; IL-18, interleukin-18; TNF-α, tumor necrosis factor-α; NF-κB, nuclear factor kappa B.

We irradiated HaCaT cells with 30 mJ/cm^2^ UVB and observed statistically significant increases in TNF-α and NF-κB/p65 but not interleukin-18 (IL-18) or E-cadherin mRNA after 24 h of incubation (*P* < 0.01, respectively) (**Figure 2A**). One microgram per milliliter tacrolimus had no direct effect on these cytokines and adhesion molecule mRNA expression in HaCaT cells; however, it could significantly inhibit UVB irradiation-induced TNF-α and NF-κB/p65 expression (*P* < 0.001, respectively) (**Figure 2A**), which was also confirmed at the protein level (**Figures 2B,C**).

### Topical Tacrolimus Inhibits High-Dose UVB Irradiation-Induced TNF-α Expression in Cultured Tissues

**FIGURE 3:**
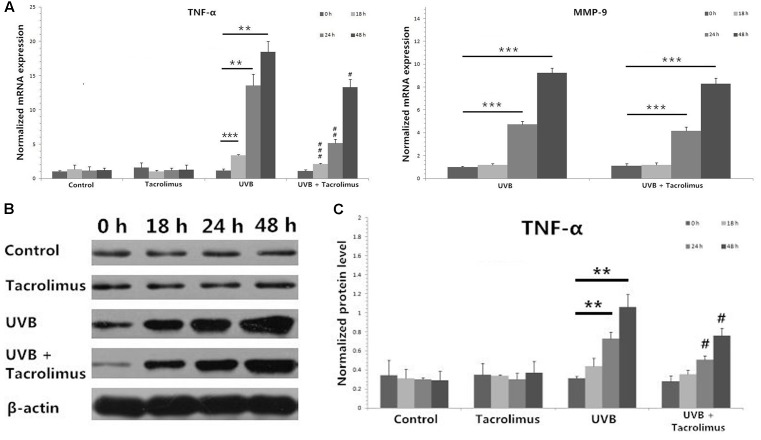
Real-time quantitative PCR analyses of cultured tissues **(A)** and protein detection by using western blot **(B,C)** at different time points. **(A)** Topical tacrolimus significantly inhibited 180 mJ/cm^2^ UVB irradiation-induced TNF-α mRNA expression in cultured tissues at 18, 24, and 48 h; however, it could not regulate UVB irradiation-induced significant increases in MMP-9 mRNA expression. **(B)** Western blot of TNF-α. **(C)** The inhibition of tacrolimus on UVB irradiation-induced TNF-α mRNA expression was confirmed at the protein level at 24 and 48 h. Statistical significance indicated: ^∗^*P* < 0.05, ^∗∗^*P* < 0.01, ^∗∗∗^*P* < 0.001, ^#^*P* < 0.05, ^##^*P* < 0.01, ^###^*P* < 0.001. Abbreviation: UVB, ultraviolet B; TNF-α, tumor necrosis factor-α; MMP-9, matrix metallopeptidase 9.

One hundred and eighty millijoules per centimeter squared UVB irradiation induced significant increases in TNF-α mRNA expression in cultured tissues at 18, 24, and 48 h (18 h, *P* < 0.001; 24 h, *P* < 0.01; 48 h, *P* < 0.01) (**Figure 3A**), which was confirmed at the protein level (24 h, *P* < 0.01; 48 h, *P* < 0.01) (**Figures 3B,C**). Topical tacrolimus could significantly inhibit high-dose UVB irradiation-induced TNF-α mRNA expression (18 h, *P* < 0.001; 24 h, *P* < 0.01; 48 h, *P* < 0.05) (**Figure 3A**), which was also confirmed at the protein level (24 h, *P* < 0.05; 48 h, *P* < 0.05) (**Figures 3B,C**). High-dose UVB irradiation also induced significant increases in matrix metallopeptidase 9 (MMP-9) mRNA expression in cultured tissues at 24 and 48 h (24 h, *P* < 0.001; 48 h, *P* < 0.001); however, it could not be regulated by topical tacrolimus (**Figure 3A**). Neither high-dose UVB single irradiation nor topical tacrolimus had direct effect on IL-1β, C–C chemokine receptor type 7 (CCR7), C–C motif ligand 19 (CCL19), and integrin alpha-6 (ITGA6) mRNA expression in tissues.

### TNF-α Antagonist Reverses High-Dose UVB Irradiation-Induced Epidermal Langerhans Cell Reduction and CD1a+ Cell Increment in Culture Medium

**FIGURE 4:**
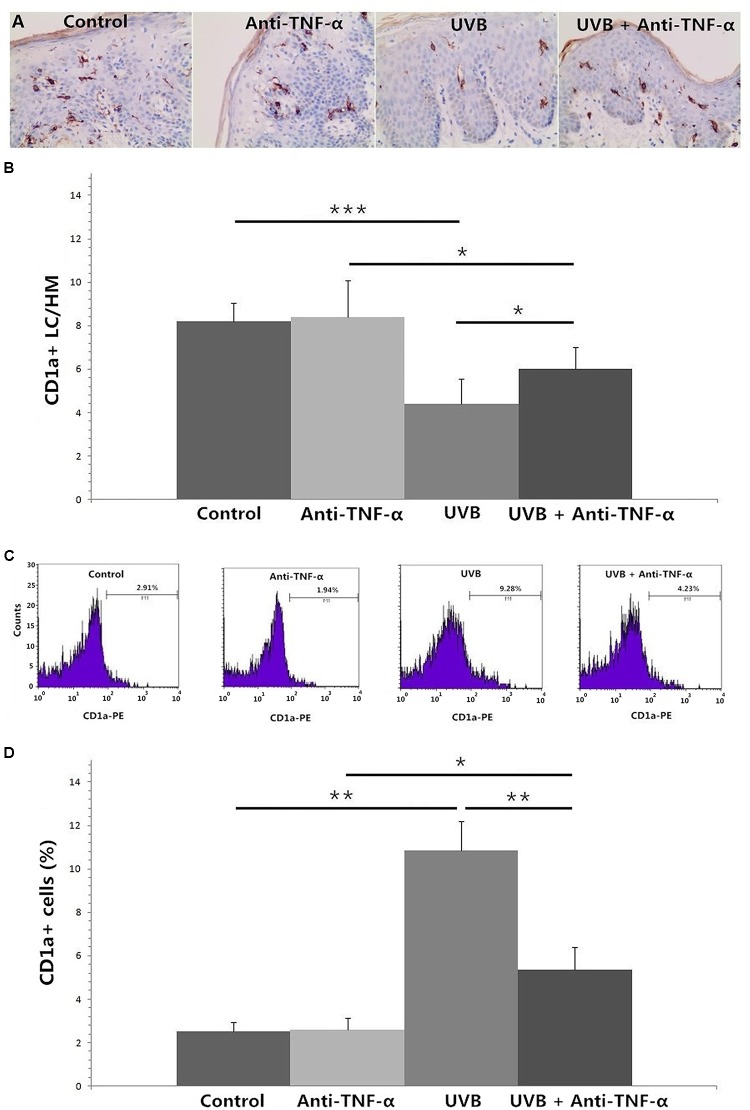
Immunohistochemical staining of CD1a in foreskin tissues (**A,B**, HM × 400) and flow cytometry of CD1a expression on cells in the culture medium **(C,D)** after 24 h of incubation. **(A)** Less CD1a+ LCs were seen in epidermis after 180 mJ/cm^2^ UVB irradiation. **(B)** TNF-α antagonist significantly reversed high-dose UVB irradiation-induced epidermal LC reduction at 24 h. **(C)** A higher percentage of CD1a+ cells in culture medium were observed in *UVB* group by using flow cytometry. **(D)** TNF-α antagonist significantly inhibited UVB irradiation-induced increment of the percentage of CD1a+ cells in culture medium. Statistical significance indicated: ^∗^*P* < 0.05, ^∗∗^*P* < 0.01, ^∗∗∗^*P* < 0.001. Abbreviation: UVB, ultraviolet; LCs, Langerhans cells; HM, high magnification; TNF-α, tumor necrosis factor-α.

**Figures 4A,B** show that TNF-α antagonist (recombinant human TNF-α receptor II: IgG Fc fusion protein, 50 μg/ml) significantly reversed high-dose UVB irradiation-induced epidermal LC reduction at 24 h (*P* < 0.05), and inhibited UVB irradiation-induced significant increment of the percentage of CD1a+ cells in culture medium (*P* < 0.01) (**Figures 4C,D**).

## Discussion

Langerhans cells represent the first line of immunological defense, which has been proved to belong to the macrophage lineage recently (Doebel et al., 2017). The proliferation, maturation, and migration of epidermal LCs can be affected by skin inflammation, and increased migration during inflammation can lead to a partial depletion of epidermal LCs. Adhesion between keratinocytes and LCs, mediated by E-cadherin, is important in the retention of LCs in the skin (Matthews et al., 2003). Epidermal LCs disengage from their adhesion with surrounding keratinocytes during the migration in part by downregulation of E-cadherin (Tang et al., 1993). Intradermal injection of antagonists against TNF-α, IL-1β, and IL-18 impairs epidermal LC migration (Cumberbatch et al., 1997, 2001). The increment of the expression of CCR7, CCL19, MMP-9, and ITGA6 also promotes LC migration from epidermis to dermis (Price et al., 1997; Randolph, 2001; Koch et al., 2006).

Previous studies observed that high-dose UVB irradiation could decrease the number of epidermal LCs, and found the possible mechanism was the increment of LC migration rather than LC apoptosis (Kölgen et al., 2002; Yin et al., 2012b). The cited study has also proved that high-dose UVB irradiation-induced epidermal LC reduction was related to LCs migration (Yin et al., 2014). Our study showed topical tacrolimus could reverse high-dose UVB irradiation-induced epidermal LC reduction by inhibiting TNF-α secretion in keratinocytes via regulation of NF-κB/p65. Anti-TNF-α treatment significantly reversed UVB irradiation-induced epidermal LC reduction and CD1a+ cell increment in culture medium. We thought the CD1a staining positive cells in the culture medium were mainly made up of migrated LCs and dermal dendritic cells, and the increased CD1a+ cells in the medium induced by UVB irradiation should most probable be LCs.

Epidermal LCs play important role in immunosurveillance, which is an important pathway against tumorigenesis. Epidermal immature LCs can discern and ingest antigen, then gradually mature during migration. Interestingly, Modi et al. (2012) reported that LCs facilitated epithelial DNA damage and squamous cell carcinoma, suggesting the complex relation between immune cells and carcinogenesis. Lewis et al. (2015) also reported that LCs could facilitate UVB-induced epidermal carcinogenesis, which was counter to the concept of LCs as key players in tumor immunosurveillance that indicated the complexity of LC functions. Transforming growth factor beta-1 (TGF-β1) signaling promoted UVB-induced skin carcinogenesis that was mediated partly through its role in UVB-induced migration of dermal dendritic cell and cutaneous inflammation (Ravindran et al., 2014).

In 2006, the US Food and Drug Administration issued “black box” warnings for TCIs due to potential safety risks including skin cancers and lymphomas, which is still lack of evidence presently (Yin et al., 2012a). Voskamp et al. (2013) reported oral tacrolimus did not enhance UV irradiation-induced skin carcinogenesis. Mitamura et al. (2011) found both topical tacrolimus 0.03% and tacrolimus 0.1% could inhibit tumor induction in a mouse model of an initiation–promotion skin tumor. Topical tacrolimus did not enhance photocarcinogenesis or induce any dermal carcinogenicity in hairless mice (Lerche et al., 2008).

Pimecrolimus cream, another kind of TCI, did not affect LC number in healthy and atopic skin (Hoetzenecker et al., 2005; Yin et al., 2012b). In this study, tacrolimus 0.03% ointment used alone has also no obvious effect on human epidermal LC number; however, topical tacrolimus reverses high-dose UVB irradiation-induced epidermal LC reduction by inhibiting LC migration via downregulation TNF-α secretion in epidermal keratinocytes. In light of the fact that LCs could facilitate UVB-induced epidermal carcinogenesis (Lewis et al., 2015), the regulation effect of tacrolimus ointment on UVB irradiation-induced LC migration would promote photocarcinogenesis or inhibit skin carcinogenesis, which needs further investigation.

## Ethics Statement

This study was carried out in accordance with the recommendations of institutional guidelines and Local Ethics Committee of the First Affiliated Hospital of Nanjing Medical University (approval number 2013-SRFA-074). All subjects gave written informed consent in accordance with the Declaration of Helsinki.

## Author Contributions

JX and YF were responsible for cell and tissue culture and qRT-PCR and western blot. GS and QG were responsible for immunohistochemistry. LY and YH were responsible for flow cytometry and data analysis. DL provided guidance to experiment design. ZY was responsible for the quality of the overall manuscript.

## Conflict of Interest Statement

The authors declare that the research was conducted in the absence of any commercial or financial relationships that could be construed as a potential conflict of interest.
